# Notes on Preparations for the Sick

**Published:** 1905-12

**Authors:** 


### Notes on Preparations for the Sick.

We are indebted to Mr. H. Wippell Gadd for the following notes on the forty-second annual meeting of the British Pharmaceutical Conference, which was held at Brighton at the end of July last.

Many of the papers contained matter of considerable interest to the medical profession.

The presidential address was an exposition of the principle of standardisation as recently applied to pharmaceutical products. Mr. Naylor said, "Standardisation, as applied to a crude product or preparation, is understood to imply that by a method of proper treatment, ascertained by practical experiments, it has been made to conform to a predetermined standard. The required standard may have a physical, chemical, or physiological basis, and may have reference either to one or more definite principles, or to a mixture of indefinable subjects, but the object of standardisation is to secure uniformity of products, more especially in respect to medicinal activity." He then proceeded to give the results of a comparative study of various processes published in recent years for the standardisation of drugs and their preparations, particularly belladonna, cinchona, ipecacuanha, nux vomica and opium. He concluded by pointing out the necessity of standards being fixed and maintained for such drugs as calabar bean, hemlock, henbane, jaborandi, stramonium and strophanthus.

Other papers which followed dealt with a variety of subjects, ranging from such ancient medicines as castor oil to the latest information concerning radio-active substances, and one on the new United States pharmacopœia showed how very great an advance it is upon anything yet published.

An interesting paper was read on the Chemistry and Pharmacy of the leaves of Viola Odorata, the author of which confirmed the discovery by mandelin of a glucoside, and showed how a solution could be prepared containing this principle freed from inert matter. No opinion was given as to the therapeutic value of the leaves, but it was thought that any activity which they possessed was due either to the glucoside, the ferment associated with it, or the product of its hydrolysis.

---

**"Artox," Whole-meal Flour and Bread.**—Appleyard's Limited, Rotherham.—The Artox flour is a pure whole meal, and being prepared by a patent process, the irritating particles of the bran are broken up—the sharp spicules of the bran, which in ordinary wheat meal are so irritating to a weak stomach, are rendered harmless. It is very well known that a whole meal is far more nourishing than the ordinary white meal, and that the ordinary white loaf is quite unable to supply all the nourishment necessary to the maintenance of human life. Many of the so-called whole meals are white flour, or seconds with a greater or smaller admixture of bran. In the Artox whole meal every particle of the wheat grain is preserved, and therefore this meal makes better bread, cakes, puddings and pastry than can be obtained by the use of white flour. It is quite true that "man cannot live by bread alone" if the bread is made from almost pure starch, but if the whole meal be used and butter added, then he may thrive on that combination without any other kind of food.

**Palatinoids.**—OPPENHEIMER, SON & CO., London.

**Trional**, gr. v., a convenient form for this slowly soluble powder.

**Cascara Compound**, a combination of cascara bark with a small dose of aloin.

**Metramine**, gr. v., a synthetical product, known chemically as hexamethylene tetramine. It has been found to be one of the best antiseptics for the genito-urinary tract.

**Compound Metramine**, contains metramine, gr. iv., with methylene blue, gr. i., and oils of copaiba and sandal.

---

**Styptol, Neutral Phthalic Acid of Cotarnine; Styracol, Guaiacol Cinnamic-acid Ether.**—KNOLL & CO., St. Mary-at-Hill, London, E.C.—These preparations, made at Ludwigshafen on Rhine by the firm of Knoll and Co., appear to be worthy of further study and trial. Much evidence is adduced in their favour, and they are being much used on the Continent.

**Styptol**, the phthalic salt of cotarnine, is said to be better than either hydrastis, ergotin, or stypticin in the treatment of uterine hemorrhage. The sedative effect on the uterus is said to be comparable with the quieting effects of opium on the intestine. In cases of hemorrhage from the uterus the effect is prompt and rarely fails. It is prepared in sugar-coated tablets, of which three to five should be taken daily, or it may be given in doses of one grain every three hours. It may be administered even during pregnancy without risk of inducing uterine contraction or labour pains. In the treatment of profuse menstruation it should be a very useful palliative. It is a fine crystalline powder, easily soluble in water, and with a somewhat bitter taste. The original package contains a tube of twenty tablets, each having 0.05 gm. (gr. $\frac{3}{4}$) of the drug.

**Styracol**, the cinnamic-acid ethereal salt of guaiacol, is insoluble in cold water, is odourless, and wholly free from any taste of guaiacol. It is believed to pass through the stomach unchanged, and in the intestine is decomposed, giving off there the two valuable antiseptics, guaiacol and cinnamic acid; of the former some 40 per cent. more is liberated than from the other guaiacol compound in common use, the guaiacol carbonate. From the guaiacol administered in the form of styracol some 86 per cent. could be obtained from the urine in combination with ethyl-sulphuric acids. It should be possible by means of this non-poisonous drug to produce a continuous impregnation of the organism of the consumptive with guaiacol. Drugs are somewhat unfashionable at present in the treatment of phthisis, but we think that this preparation is one which should have an extended and persevering trial in all forms of tuberculosis. It may be given in powder with an equal quantity of sugar, or in tablets each containing half a gramme. The daily dose may be increased to two drachms.

---

**Tabloid.**—BURROUGHS, WELLCOME & Co.

**Rhubarb Extract,** gr. ij.—This preparation is made by a special process whereby the full therapeutic value of the drug is retained. It is a valuable stomachic and purgative, stimulating the flow of gastric juice and increasing peristalsis. In the digestive ailments of children it should be most efficient and acceptable.

One to three tabloids should be swallowed with a little water after food as may be deemed needful.

---

**Pleated and Compressed Bandages and Dressings, "Tabloid" Brand.**—BURROUGHS, WELLCOME & Co.—We have received samples of bandages, boric lint, sal alembroth gauze, cotton wool, and other dressings, packed in such small parcels that a considerable quantity can be carried in the coat pocket. The pleating of the dressings permits them to be easily unfolded, and their coverings of parchment paper and tinfoil keep them from contamination. These compressed dressings should be very useful to the general practitioner.

---

**Fer Bravais.**—130 Rue Lafayette, Paris.—A sample of this well-known preparation has been forwarded to us. It has been in use many years, and has at many exhibitions received gold, silver, and other medals with diplomas of distinction. In a concentrated form it possesses neither taste nor smell, and it can be diluted with wine, water, or other liquid without precipitation. It is a solution of peroxide of iron in water, so that it is in fact a soluble hydrate of iron, possessing all the good qualities of iron for medicinal purposes with none of its disadvantages. It has by its merits won considerable renown, and is likely to continue to be largely used.

---

**Allenbury Diet.**—ALLEN & HANBURY, Plough Court, London, E.C.—The popularity of the various "Allenbury" infant foods has justified the makers in bringing the Allenbury Diet before the public. It is especially prepared for adult use, where a readily assimilable yet nourishing food is required. Its basis is farinaceous, but pancreatised dried milk is also included in its composition. One feature worthy of special notice is its freedom from excessive sweetness, so often a great drawback in invalids' foods. The readiness with which it can be prepared—simply by the addition of boiling water—should also commend it for use in the sick room.

**Yohimbine Spiegel.** — Chemische Fabrik Güstrow, Drs. Hillringhans and Heilmann; H. W. Brummerstaedt & Co., 3 Cross Lane, St. Mary-at-Hill, London, E.C.—The substance bearing this name is a well-defined crystalline alkaloid, first isolated by Dr. L. Spiegel from yohimbehe bark, which comes from a tree belonging to the natural order Rubiaceæ.

According to Ludwig Scholz decoctions of this bark have long been used as a tonic by natives of the Cameroons.

Its physiological activity has been experimentally determined by administering it to numerous animals, and it has a very marked effect on the sexual organs, being a powerful vaso-dilator. Yohimbine Spiegel appears to stimulate the lumbar region of the spinal cord, and the action has been observed to be curative in some cases of spinal weakness. The preparation should prove to be one of the most reliable aphrodisiacs; it has also been observed to possess a local anæsthetic action when applied to mucous membranes, but its toxicity is far less than that of cocaine.

---

**Iothion** (Bayer).—The Bayer Co. Ltd., 19 St. Dunstan's Hill, London, E.C.—This is a new organic preparation of iodine, intended for both internal and external use. Chemically it is a derivative of hydroxy-propane, and the therapeutic activity of the aliphatic halogen substitution products is too well known to need any comment. Iothion contains about 80 per cent. iodine, and is thus equal in strength to iodide of potassium, but its composition renders it more liable to absorption than inorganic compounds of iodine, and it does not give rise to irritation. In the form of an ointment it is best to use it combined with anhydrous lanoline, with or without the addition of a little vaseline, the latter substance rendering the unguent of more suitable consistency. Clinical experiments show that Iothion, applied externally, is often superior to the iodides usually administered internally, especially in cases of tabes and syphilitic neuritis.

---

**Histogénol** (Naline).—Roberts & Co., 76 New Bond Street, London, W.—Histogénol is described as an active reconstituant for use in wasting diseases. It contains organically combined phosphorus and arsenic, and is prepared in the form of emulsion, elixir, and granules, also in bulbs for hypodermic injections.

From a physiological and chemical standpoint organically combined medicaments should possess greater therapeutic activity than when present as inorganic compounds, and numerous clinical observations tend to prove this assumption in the case of Histogénol.

It is recorded that in many cases the use of this preparation is followed by increase of weight, restoration of nitrogenous equilibrium, and normal respiratory exchange. Such statements justify a trial in cases where a reconstituant is indicated. The makers claim that this remedy is capable of powerfully combating the evolution of pulmonary tuberculosis.

---

**Brand's Beef Tea Tabules.**—Brand & Co. Ltd., Mayfair, W.—These tabules are put up for sale in boxes of one dozen, one tabule being sufficient to make a breakfast-cupful of beef tea. A chemical examination shows that they contain proteid, an important constituent of all preparations used not only as stimulants but as actual foods. The flavour of the finished product is also agreeable, and we can with confidence recommend their use.

---

**Metakalin** (Bayer).—Metakalin is a readily soluble antiseptic, which owes its efficacy to the presence of meta-cresol. The three isomeric cresols have long been in use as disinfectants, but it is believed that the meta compound is the most active. The toxicity of meta-cresol is less than that of phenol, but its antiseptic properties are probably equal. Metakalin is supplied in small cartridges, also in the form of tablets.

